# The plant metabolome guides fitness-relevant foraging decisions of a specialist herbivore

**DOI:** 10.1371/journal.pbio.3001114

**Published:** 2021-02-18

**Authors:** Ricardo A. R. Machado, Vanitha Theepan, Christelle A. M. Robert, Tobias Züst, Lingfei Hu, Qi Su, Bernardus C. J. Schimmel, Matthias Erb

**Affiliations:** 1 Institute of Plant Sciences, University of Bern, Bern, Switzerland; 2 Institute of Biology, University of Neuchâtel, Neuchâtel, Switzerland; 3 Institute of Soil and Water Resources and Environmental Science, College of Environmental and Resource Sciences, Zhejiang University, Hangzhou, China; 4 Zhejiang Provincial Key Laboratory of Agricultural Resources and Environment, Zhejiang University, Hangzhou, China; Sainsbury Laboratory, UNITED KINGDOM

## Abstract

Plants produce complex mixtures of primary and secondary metabolites. Herbivores use these metabolites as behavioral cues to increase their fitness. However, how herbivores combine and integrate different metabolite classes into fitness-relevant foraging decisions in planta is poorly understood. We developed a molecular manipulative approach to modulate the availability of sugars and benzoxazinoid secondary metabolites as foraging cues for a specialist maize herbivore, the western corn rootworm. By disrupting sugar perception in the western corn rootworm and benzoxazinoid production in maize, we show that sugars and benzoxazinoids act as distinct and dynamically combined mediators of short-distance host finding and acceptance. While sugars improve the capacity of rootworm larvae to find a host plant and to distinguish postembryonic from less nutritious embryonic roots, benzoxazinoids are specifically required for the latter. Host acceptance in the form of root damage is increased by benzoxazinoids and sugars in an additive manner. This pattern is driven by increasing damage to postembryonic roots in the presence of benzoxazinoids and sugars. Benzoxazinoid- and sugar-mediated foraging directly improves western corn rootworm growth and survival. Interestingly, western corn rootworm larvae retain a substantial fraction of their capacity to feed and survive on maize plants even when both classes of chemical cues are almost completely absent. This study unravels fine-grained differentiation and combination of primary and secondary metabolites into herbivore foraging and documents how the capacity to compensate for the lack of important chemical cues enables a specialist herbivore to survive within unpredictable metabolic landscapes.

## Abstract 2

This study shows how an important agricultural pest, the western corn rootworm, integrates nutrients (sugars) and plant defense compounds (benzoxazinoids) to navigate the maize rhizosphere and feed on the most nutritious roots.

## Introduction

Herbivore foraging behavior contributes to the distribution and performance of herbivores and plants in natural and agricultural ecosystems [1–3]. Insect herbivores often exhibit pronounced oviposition and feeding preferences for specific plant species, genotypes within species physiological states within genotypes [1,4,5]. Most insect herbivores also show characteristic preferences for specific plant organs and tissues [6–8].

Herbivores establish preferences through different types of host cues [9–12]. Chemical cues, including plant primary and secondary metabolites, are particularly important for herbivores, as they provide specific information about the identity, physiological status, and nutritional value of host plants and tissues [13–15]. The overarching view in the field of chemical ecology is that primary metabolites are used as cues to identify nutritious hosts and tissues, while volatile and nonvolatile secondary metabolites are used as indicators of toxicity and defense status, and as signature cues of specific host plant lineages and species [16–18]. Specialized herbivores in particular are often attracted and stimulated by host-specific secondary metabolites [19]. Over the past decades, herbivores have been found to respond to a multitude of plant primary and secondary metabolites in artificial diet experiments [13,16,17]. An increasing number of studies now also document the importance of these metabolites in planta through molecular manipulative approaches [20–26].

Plants produce diverse sets of primary and secondary metabolites [27], and herbivores likely combine many of these metabolites into their foraging behavior [16,28,29]. Chemical cue integration is thought to allow herbivores to obtain more accurate information about the nutritional value and toxicity of complex host plant metabolomes [17] and thus to increase the robustness of their foraging decisions [12,29]. Many insect herbivores are known to be attracted to specific combinations of volatile chemicals [14,29]. Furthermore, some herbivores avoid combinations of secondary metabolites more strongly than individual compounds [30–32]. A limited number of studies also indicate that herbivores may be able to integrate primary and secondary metabolites into their foraging strategies [17]. In artificial diet experiments, tannins reduce food intake by locusts at low protein:carbohydrate ratios, but not at high ratios, a behavior which mirrored the conditional impact of tannins on locust performance [33,34]. Prior exposure to secondary metabolites modulate subsequent food choice in both insect herbivores [35] and mammals [36,37]. Conversely, sugars mask the aversive taste of secondary metabolites [38]. Despite these advances, we currently lack a detailed understanding of how primary and secondary metabolites interact to determine herbivore behavior under biologically realistic conditions [9]. The paucity of manipulative experiments that test for interactions between host-derived chemical foraging cues in planta limits our capacity to assess the concerted impact of different metabolites on herbivore feeding preferences, and, more generally, our understanding of the role of plant metabolic complexity in herbivore behavior and plant-herbivore interactions.

An implicit assumption of herbivore foraging theory is that herbivore behavior improves herbivore fitness [39]. With some notable exceptions, herbivores generally prefer to oviposit and feed on plants and tissues that increase their performance [40–42]. Host plant chemicals likely play an important role in these preference–performance relationships [12,17]. However, the contribution of individual behavioral cues to herbivore fitness in the context of the full chemical complexity of a given host plant has remained difficult to quantify [30,43,44]. Targeted molecular manipulation of the production and perception of chemical cues provides new opportunities in this context, including the evaluation of the benefits of the integration of multiple chemical cues into herbivore foraging [29], and the assessment of the importance of the flexible use of multiple foraging cues with redundant information content [11,12].

Insect herbivores use chemosensory receptors to detect volatile and nonvolatile plant chemicals [45,46]. Gustatory receptors (GRs) are required for the detection of a variety of chemicals [47–49], including nonvolatile plant chemicals such as alkaloids [24] and sugars [50]. Knocking out specific GRs reduces oviposition of swallowtail (*Papilio xuthus*) butterflies [24] and host recognition by silkworm (*Bombyx mori*) larvae [23], thus demonstrating the functional importance of individual GRs for herbivore behavior. Work in fruit flies (*Drosophila melanogaster*) revealed that some GRs are broadly tuned and can mediate avoidance to many different compounds [51], while others are narrowly tuned and confer responsiveness to specific compounds [52]. Gr43a is a highly conserved insect taste receptor that specifically responds to D-fructose in Drosophila [49], the diamondback moth (*Plutella xylostella*) [53], and the cotton bollworm (*Helicoverpa armigera*) [54]. As Gr43a is responsible for the detection of D-fructose in the hemolymph as a proxy for carbohydrate supply, *Gr43a*-silenced flies also become unresponsive to other dietary sugars [55]. Interestingly, different GRs can interact dynamically through competition, inhibition, and activation [47,56,57], resulting in substantial potential for GR-mediated integration of multiple chemical cues into behavioral responses.

Here, we developed a manipulative approach to evaluate the importance of maize primary and secondary metabolites for the foraging and foraging-dependent performance of the western corn rootworm (*Diabrotica virgifera virgifera*). Maize is a major global crop plant that produces a variety of chemical defenses, including benzoxazinoids [58], phenolic acid derivatives [59], and diverse volatile and nonvolatile terpenoids [60–63]. The western corn rootworm is an economically damaging maize pest [64]. Adults are polyphagous, but typically feed on maize before laying their eggs into the soil of maize fields in late summer [65]. Larvae hatch in spring, synchronized with the growth of newly sown maize. In contrast to adults, western corn rootworm larvae are highly specialized on maize roots [65]. Over the last decades, the chemical ecology of the western corn rootworm has been studied in detail [13,64,65]. Field and laboratory studies revealed tight associations between maize root chemistry and western corn rootworm behavior. Western corn rootworm larvae respond behaviorally to a wide variety of chemical cues, including CO_2_ [66], sugars and fatty acids [67,68], aromatic and terpene volatile organic compounds [69,70], conjugated phenolic acids [71], and benzoxazinoids [72]. The emerging picture is that these chemicals likely allow western corn rootworm larvae to locate plant roots from a distance [73], to discriminate between plants of different quality [69–71,74], and to identify and feed on the most nutritious roots [72,75]. Several in vitro experiments also suggest that the western corn rootworm can combine multiple chemical cues for host finding and acceptance [67], hinting at the substantial sensory capacity of this specialist root feeder. Here, we used a metabolomics approach to identify potential nonvolatile cues that guide root finding and acceptance by western corn rootworm larvae. We then independently manipulated the availability of 2 classes of identified candidate feeding cues, benzoxazinoids and sugars, for the western corn rootworm. Through a combination of plant genetics and insect RNA interference (RNAi), we demonstrate that sugars and benzoxazinoids serve both specific and combined roles as determinants of the behavior and behaviorally driven performance of this specialist herbivore.

## Results

### The western corn rootworm prefers to feed on root tissues that are rich in benzoxazinoids and soluble sugars

**Fig 1:**
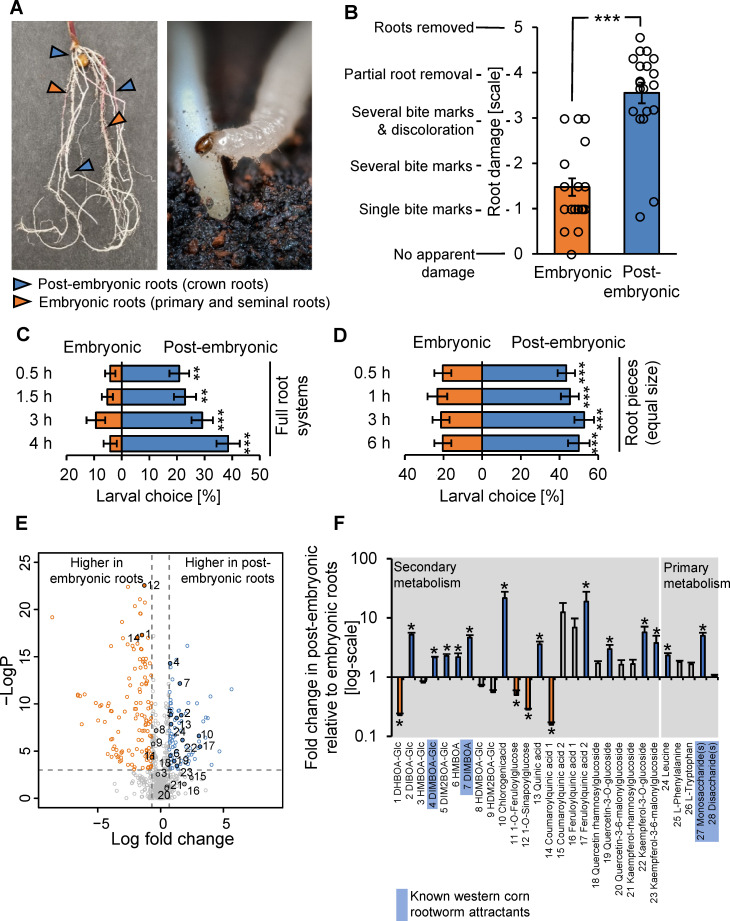
The western corn rootworm prefers to feed on root tissues that are rich in benzoxazinoids and soluble sugars. (A) Left: Young maize plants produce embryonic primary and seminal roots (orange arrows) and postembryonic crown roots (blue arrows). Right: Western corn rootworm larvae are highly specialized maize root feeders that are very mobile in the second and third instar. A third instar larvae is shown. (B) Average damage score observed on postembryonic and embryonic roots of soil-grown maize plants after 7 days of infestation by western corn rootworm larvae (***p < 0.001, Wilcoxon Signed Rank test, *n* = 20 plants with 15 larvae each, data from Fig 4D). Dots represent damage scores on individual plants (averaged within root types). For frequency distributions of individual roots, refer to S1 Fig. (C) Preference of western corn rootworm larvae for postembryonic and embryonic roots within the root system (**p < 0.01, ***p < 0.001, FDR-corrected Least Square Mean post hoc tests, *n* = 16 dishes with 6 larvae each). (D) Preference of western corn rootworm larvae for root pieces of equal size of postembryonic and embryonic roots (***p < 0.001; FDR-corrected Least Square Mean post hoc tests, *n* = 18 dishes with 5 larvae each). (E) Metabolomics profiles of methanolic extracts of postembryonic and embryonic roots. Orange features are more abundant in embryonic roots, blue features are more abundant in postembryonic roots (min. 2-fold difference, *p* < 0.05, FDR-corrected Student *t* tests, *n* = 9–10). Numbers denote features that were tentatively assigned to structures based on exact mass and fragment information (S1 Table). (F) Relative abundance differences of tentatively identified metabolites between postembryonic and embryonic roots (fold change of peak areas; *p < 0.05, FDR-corrected Student *t* tests, *n* = 9–10). Error bars denote SEM. Underlying data can be found in S1 Data. FDR, false discovery rate; SEM, standard errors of means. *Picture credits*: *Ricardo Machado*, *Lingfei Hu*, *Cyril Hertz*.

The root system of young maize plants consists of embryonic and postembryonic roots (Fig 1A). Embryonic roots emerge directly from the embryo and comprise primary and seminal roots. Postembryonic roots emerge from the hypocotyl and stem and comprise crown roots, and, at later developmental stages of the plant, internode-derived brace roots [76]. To determine feeding preferences of the western corn rootworm within the root system of young maize plants, we infested soil-grown maize plants with western corn rootworm larvae for 7 days and then scored the damage on the different root types. In line with earlier studies [72,75], we found low amounts of damage on embryonic roots and substantial damage on postembryonic roots (Fig 1B). While embryonic roots showed scattered bite marks, postembryonic roots were often partially or even fully removed (S1 Fig). To test whether this feeding preference is reflected in the distribution of the larvae within the root system, we carried out a series of behavioral experiments. First, we laid out intact maize root systems on a filter paper and then recorded the position of second instar larvae at different time points after their release. Five times more larvae were found on postembryonic than embryonic roots 30 min after the release of the larvae (Fig 1C). This preference persisted over the duration of the experiment. To test whether the preference of the larvae for postembryonic roots may be due to differences in abundance or the relative position of the 2 root types within the root system (Fig 1A), we offered postembryonic and embryonic root pieces of equal size to the larvae. Similar to what was observed for entire root systems, significantly more larvae were found on the postembryonic root pieces than the embryonic root pieces after 30 min (Fig 1D). Thus, the western corn rootworm preferentially stays and feeds on postembryonic roots of young maize plants.

To gain insights into the phytochemical differences that may drive the preference of the western corn rootworm for postembryonic roots, we performed untargeted metabolomics on methanolic extracts of both root types. Using UHPLC-Q-TOF-MS and ESI-, 4512 total mass features were detected, corresponding to 1,563 informative features (Fig 1E). Of these, 81 and 137 features were enriched in postembryonic or embryonic roots of wild-type (WT) plants, based on more than 2-fold difference in normalized signal intensity at a statistical significance of *p* < 0.05 (Fig 1E). Using exact masses and fragmentation patterns, 28 metabolites could be putatively identified within the dataset. Identified metabolites included benzoxazinoids, phenolic acid derivatives, amino acids, and sugars (Fig 1F, S1 Table). Seventeen of these metabolites showed root-type–specific accumulation patterns (Fig 1F). Among the secondary metabolites, benzoxazinoids and phenolic acids showed pronounced shifts in their profiles, with some metabolites accumulating in higher amounts in postembryonic roots and others accumulating in higher amounts in embryonic roots (Fig 1F). Among the detected primary metabolite features, amino acids and sugars were more abundant in postembryonic than in embryonic roots, with the most pronounced shifts observed for features matching leucine and monosaccharides (Fig 1F). Thus, the preference of the western corn rootworm for postembryonic roots is associated with distinct primary and secondary metabolite accumulation patterns in these tissues.

To narrow down the list of metabolites that may prompt the western corn rootworm to feed on postembryonic roots, we performed a literature search on western corn rootworm attractants and feeding stimulants. Thirteen different compounds known to elicit behavioral responses in the western corn rootworm in vitro were found (S2 Table). Cross-referencing this table with the list of chemicals that accumulated in postembryonic roots at higher concentrations resulted in the identification of 4 candidate compounds that may mediate postembryonic root preference: the monosaccharides glucose and fructose, which act as feeding stimulants [67], and the benzoxazinoids DIMBOA-Glc and DIMBOA, which form iron complexes in the rhizosphere that act as short-distance host preference and acceptance cues [72]. Based on these results and earlier studies [72,75], we hypothesized that benzoxazinoids and monosaccharides may interact to determine the preference of the western corn rootworm for postembryonic roots. Given the observed overlap between postembryonic root preference and overall plant attractiveness [72], we also hypothesized that the same compounds may mediate general host recognition and acceptance in this specialist herbivore.

### Independent manipulation of benzoxazinoids and sugars as foraging cues

**Fig 2:**
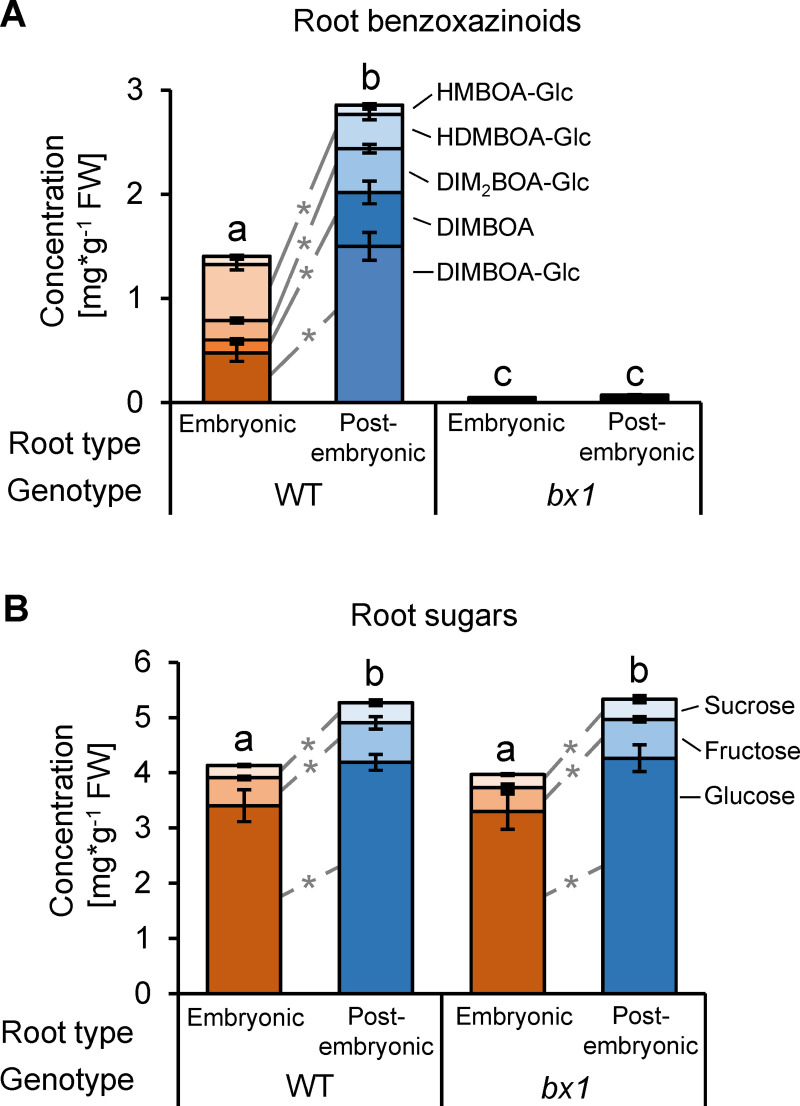
A mutation in the Bx1-gene suppresses root-type–specific benzoxazinoid accumulation independently of root sugars. (A) Concentrations of benzoxazinoids in embryonic and postembryonic roots of WT B73 and *bx1* mutant plants (*n* = 11–18). For an expanded panel of residual benzoxazinoid concentrations in the *bx1* mutant, see S2 Fig. (B) Concentrations of glucose, fructose, and sucrose in embryonic and postembryonic roots of WT B73 and *bx1* mutant plants (*n* = 8). Letters indicate significant differences in total amounts between root types and genotypes (*p* < 0.05, Holm–Sidak post hoc tests). Asterisks indicate significant differences in the concentrations of individual compounds between postembryonic and embryonic roots (*p* < 0.05, Holm–Sidak post hoc tests). Error bars denote SEM. Underlying data can be found in S1 Data. SEM, standard errors of means; WT, wild-type.

To test for the individual and combined roles of benzoxazinoids and sugars in mediating western corn rootworm behavior, we sought to independently manipulate their availability as foraging cues. The first dedicated step in benzoxazinoid biosynthesis is mediated by the indole-3-glycerol phosphate lyase *Bx1* [58]. By consequence, *bx1* mutants are benzoxazinoid deficient [58,77]. To test if *bx1* mutant plants can be used to manipulate benzoxazinoids independently of root sugars, we performed a series of targeted analyses of WT B73 and *bx1* roots. In accordance with our metabolomics screen and earlier studies [72,75], postembryonic roots of WT plants contained 4-fold higher DIMBOA levels and 2- to 3-fold higher DIMBOA-Glc and DIM_2_BOA-Glc levels, but lower HDMBOA-Glc levels than embryonic roots (Fig 2A). Benzoxazinoid levels in the *bx1* mutant were reduced by more than 97%, and residual benzoxazinoid concentrations were not significantly different between embryonic and postembryonic roots (Fig 2A and S2 Fig). As expected from the metabolomic analyses, postembryonic roots of WT plants contained higher levels of the monosaccharides glucose and fructose than embryonic roots (Fig 2B). Sucrose levels were also higher in postembryonic roots (Fig 2B). Sugar profiles were similar in WT and *bx1* roots (Fig 2B), demonstrating that benzoxazinoid availability can be manipulated independently of sugar availability. The differential abundance between and embryonic and postembryonic roots of the other 17 identified metabolic features was conserved in the *bx1* mutant as well (S3 Fig). We only detected a significant interaction between root type and genotype for tryptophan, which is also derived from indole-3-glycerol phosphate [58]. Tryptophan accumulated in higher amounts in postembryonic roots of both WT and *bx1* mutant plants, and this difference was more pronounced in the *bx1* mutant (S3 Fig). For 8 of the 17 compounds, we also found a significant genotype effect. Phenolic compounds, including several flavonoids, showed small differences in abundance in the *bx1* mutant compared to WT plants (S3 Fig). To further explore potential metabolomic differences between embryonic and postembryonic roots of *bx1* mutant and WT plants, we tested all 1,563 informative mass features for statistically significant interactions between root type and genotype using linear models. After false-positive correction and excluding putatively identified compounds, we found 47 predominantly minor features that showed a significant interaction at *p* < 0.05. We also found 244 additional features that differed significantly between WT and *bx1* mutant roots, but again many of these were only present at low abundances. Thus, while benzoxazinoids represent the major metabolic difference between WT and *bx1* mutant roots, other metabolic features also differ between the genotypes and between root types within genotypes. Thus, we performed an additional experiment to evaluate the utility of the *bx1* mutant to test for the specific role of benzoxazinoids in mediating western corn rootworm foraging by complementing postembryonic roots of the *bx1* mutant with Fe(III)(DIMBOA)_3_ and observing larval feeding preferences. As expected, the western corn rootworm lost its preference for postembryonic roots in the *bx1* mutant (S4 Fig). Applying Fe(III)(DIMBOA)_3_ was sufficient to reestablish the preference for postembryonic roots (S4 Fig), thus justifying the use of this genotype to assess the behavioral impact of benzoxazinoids on western corn rootworm behavior.

**Fig 3:**
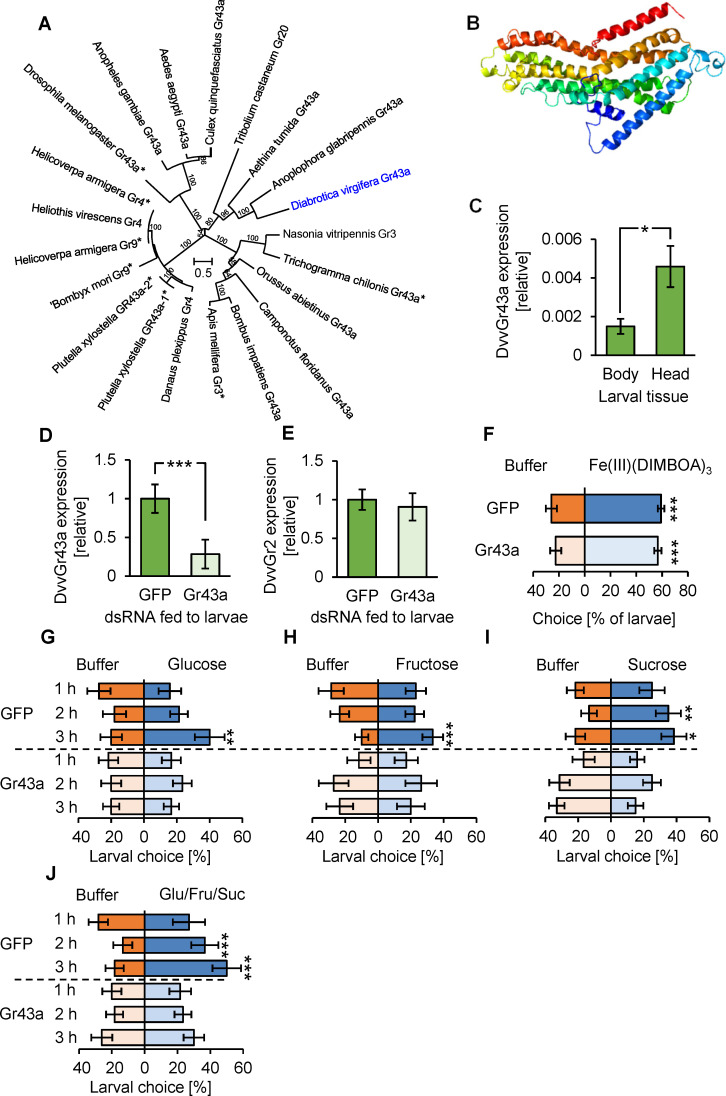
*DvvGr43a* mediates sugar preference of the western corn rootworm without influencing responsiveness to benzoxazinoids. (A) Phylogenetic relationships between gustatory sugar receptors of different insects and *DvvGr43a* of the western corn rootworm. The tree is based on protein sequences and drawn to scale, with branch lengths measured in the number of substitutions per site. Asterisks indicate functionally characterized receptors. (B) Protein tertiary structure of *DvvGr43a* as predicted with the Phyre2 algorithm. (C) *DvvGr43a* expression in the bodies (thorax and abdomen) or heads of second instar western corn rootworm larvae (*p < 0.05, Student *t* test *n* = 10). (D) *DvvGr43a* expression in western corn rootworm larvae fed with dsRNA targeting GFP (control) or *DvvGr43a* (Gr43a, ***p < 0.001, Student *t* test, *n* = 11). (E) *DvvGr2* expression in western corn rootworm larvae fed with dsRNA targeting GFP (control) or *DvvGr43a* (*n* = 8). (F) Preference of GFP and *DvvGr43a* dsRNA fed larvae for buffer or Fe(III)(DIMBOA)_3_ on filter discs after 1 hour (***p < 0.001, FDR-corrected Least Square Mean post hoc tests, *n* = 10 dishes with 6 larvae each). (G–J) Preference of GFP or *DvvGr43a* dsRNA fed larvae for buffer or glucose, fructose, sucrose, or a mixture of the three on filter discs at different time points (*p < 0.05; **p < 0.01; ***p < 0.001, FDR-corrected Least Square Mean post hoc tests, *n* = 15 dishes with 6 larvae each). Error bars denote SEM. Underlying data can be found in S1 Data. dsRNA, double-stranded RNA; FDR, false discovery rate; GFP, green fluorescent protein; SEM, standard errors of means.

To manipulate the availability of sugars as foraging cues independently of benzoxazinoids, we targeted sugar perception in the western corn rootworm. This approach was chosen because manipulation of primary metabolism in plants, including sugar complementation, results in pleiotropic effects [78,79]. Sugar perception in insects is highly conserved and mediated by *Gr43a*-like genes [49,50,53,54,80–83]. BLAST search of publicly available nucleotide sequences revealed a single putative *Gr43a* gene in the western corn rootworm, here named *DvvGr43a* (Fig 3A). The full-length sequence of this gene can be retrieved from NCBI [Accession: XM_028279548.1]. Similarity between *DvvGr43a* and putative *Gr43a* genes from different insects, many of which have been functionally characterized, was found to be between 42% and 64% at the protein level. No other closely related genes were found in western corn rootworm genome. Structural prediction indicated that the *DvvGr43a* gene encodes a seven-transmembrane domain protein, consistent with its putative role as a GR (Fig 3B). Gene-expression profiling revealed stronger expression of *DvvGr43a* in the head than the body of western corn rootworm larvae (Fig 3C). To manipulate *DvvGr43a*, we targeted its expression through RNAi by feeding the larvae with a 240-bp double-stranded RNA (dsRNA) targeting *DvvGr43a*. Compared to control larvae that were fed with dsRNA targeting green fluorescent protein (GFP), this treatment resulted in a >70% reduction in *DvvGr43a* transcript abundance (Fig 3D). To assess silencing specificity, we measured the transcription of the putative CO_2_ receptor *DvvGr2* [73]. Feeding dsRNA targeting *DvvGr43a* did not change the transcript abundance of *DvvGr2* (Fig 3E). Accordingly, the responsiveness of *DvvGr43a*-silenced larvae to volatile CO_2_ remained intact (S5 Fig). To evaluate whether *DvvGr43a* silencing changes the ability of western corn rootworm to respond to benzoxazinoids, we conducted choice assays with the behaviorally active benzoxazinoid iron complex Fe(III)(DIMBOA)_3_ [72]. After 1 hour, the majority of western corn rootworm larvae were found on filter paper discs spiked with Fe(III)(DIMBOA)_3_, independently of *DvvGr43a* silencing (Fig 3F). Next, we tested whether *DvvGr43a* silencing changes the ability of the larvae to respond to sugars. Three hours after the start of the experiment, control larvae preferred to locate on filter discs spiked with glucose, fructose, sucrose, or a mixture of the 3 compounds at concentrations of 30 mg*ml^−1^ (Fig 3G–3J). These preferences were absent in larvae fed with *DvvGr43a* dsRNA, demonstrating that silencing *DvvGr43a* eliminates the capacity of western corn rootworm larvae to respond behaviorally to 3 major plant sugars at concentrations of 30 mg*ml^−1^ (Fig 3G–3J). *DvvGr43a*-dependent sugar preference was similar in nonstarved and starved western corn rootworm larvae (S6 Fig). An additional dose-response experiment with sugar mixtures revealed a preference of WT western corn rootworm larvae to concentrations between 3 and 100 mg*ml^−1^ glucose, fructose, and sucrose. No preference was observed for *DvvGr43a*-silenced larvae, apart from the highest concentration that was tested (100 mg*ml^−1^), for which a preference was observed, most likely because of residual *DvvGr43a* activity in the silenced larvae (S7 Fig). Postembryonic maize roots contain sugars at individual concentrations between 2 and 5 mg*g^−1^ fresh weight [75], which is well below the response threshold of *DvvGr43a*-silenced larvae. Thus, with some limitations, combining *bx1* plant mutants and *DvvGr43a*-silenced larvae can be used to evaluate the individual and combined impact of benzoxazinoids and physiological levels of sugars on herbivore foraging.

### Benzoxazinoids and sugars play distinct roles in root herbivore foraging

**Fig 4:**
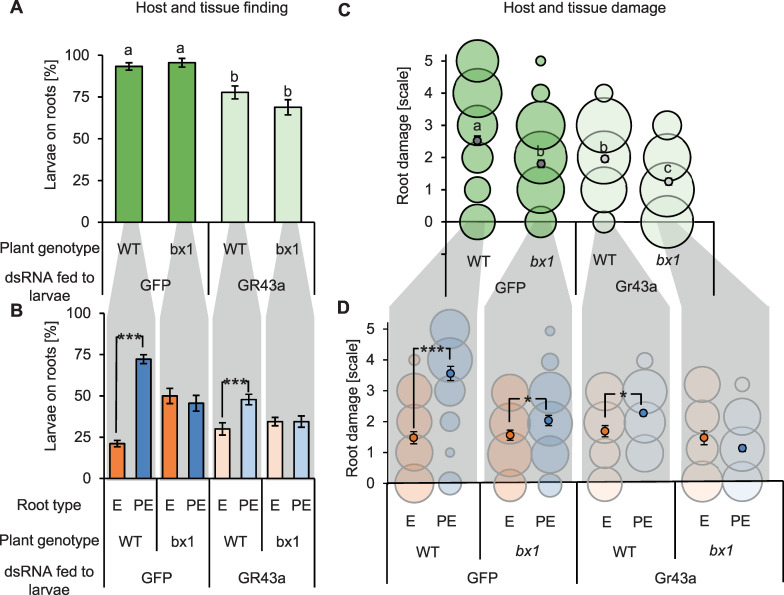
Benzoxazinoids and sugars play distinct roles in root herbivore foraging in vivo. (A) Proportion of control (GFP) or *DvvGr43a*-silenced (Gr43a) larvae found on the roots of WT B73 or *bx1* mutant roots 4 hours after their release. Letters indicate statistically significant differences between treatments (Holm–Sidak post hoc tests, *p <* 0.05, *n* = 15 dishes with 6 larvae each). (B) Proportion of control (GFP) or *DvvGr43a*-silenced (Gr43a) larvae found on embryonic (E) and postembryonic (PE) roots within the same experiment. Asterisks indicate significant differences between root types (***p < 0.001, FDR-corrected Least Square Mean post hoc tests, *n* = 15 dishes with 6 larvae each). (C) Average feeding damage by control (GFP) or *DvvGr43a*-silenced larvae per root of soil-grown WT B73 or *bx1* mutant plants 7 days after infestation. Letters indicate statistically significant differences (*p <* 0.05, Tukey post hoc tests, *n* = 20 plants with 15 larvae each). Bubble plots are shown for illustrative purposes. The sizes of the circles are proportional to the relative frequency (% within each treatment) of the different types of observed damage (each column sums up to 100%). For damage scale, refer to Fig 1B. (D) Average feeding damage on embryonic (E) or postembryonic (PE) roots within the same experiment. Asterisks indicate significant differences in root damage between root types (*p < 0.05, ***p < 0.001 Wilcoxon Signed Rank tests, *n* = 20 plants with 15 larvae each). Bubble plots are shown for illustrative purposes. The sizes of the circles are proportional to the relative frequency (% within each root type) of the different types of observed damage (each column sums up to 100%). Error bars denote SEM. Underlying data can be found in S1 Data. E, embryonic; FDR, false discovery rate; GFP, green fluorescent protein; PE, postembryonic; SEM, standard errors of means; WT, wild-type.

To understand the importance of benzoxazinoids and sugars in mediating host and tissue finding of the western corn rootworm in a neutral environment, we observed the behavior and feeding of control and *DvvGr43a*-silenced larvae on WT and *bx1* mutant root systems laid out on moist filter paper using a full-factorial design. More than 90% of control larvae were found on the roots rather than on the filter paper 4 hours after their release (Fig 4A). This number dropped to about 70% for *DvvGr43a*-silenced larvae, independently of plant genotype. A different pattern was found when looking at the distribution of the larvae on the different root types within root systems (Fig 4B). On WT plants, significantly more control larvae were found on postembryonic than embryonic roots. This difference was absent for control larvae feeding on *bx1* mutant plants. For *DvvGr43a*-silenced larvae, more larvae were found on postembryonic than embryonic roots, even though this preference was attenuated relative to control larvae. No preference was found for *DvvGr43a*-silenced larvae feeding on *bx1* mutant roots (Fig 4B). From these results, we infer that sugars contribute to root system finding and postembryonic root preference but are not strictly required for either process. Benzoxazinoids on the other hand do not seem to contribute to root system finding but are specifically required for the capacity of the western corn rootworm to distinguish postembryonic from embryonic roots.

To evaluate whether benzoxazinoids and sugars affect host acceptance in the form of active feeding, we infested roots of soil-grown WT and *bx1* mutant plants with control or *DvvGr43a*-silenced larvae and inspected the roots for characteristic feeding marks and damage after 7 days. As feeding by the western corn rootworm cannot be observed directly in the soil, and root biomass is a poor proxy for root consumption due to confounding root regrowth effects and the disproportionate impact of severed roots [84], this approach was considered most suitable for the question at hand. The experiment revealed that root damage across different roots was increased with the availability of benzoxazinoids and sugars as foraging cues in an additive fashion (Fig 4C). Frequent root removal was observed in WT plants infested with control larvae. Intermediate root damage in the form of frequent biting marks and brown discoloration was observed for *bx1* mutant roots infested with control larvae and WT roots infested with *DvvGr43a*-silenced larvae. Infrequent biting marks and/or no visible damage was observed for *bx1* mutant roots infested with *DvvGr43a*-silenced larvae. The same additive pattern was also visible when looking at the distribution of damage patterns between the different root types (Fig 4D). Postembryonic root damage was strongest and significantly more pronounced than embryonic root damage in WT plants infested with control larvae. Postembryonic root damage and damage differences with embryonic roots were intermediate for *bx1* mutants infested with control larvae and WT plants infested with *DvvGr43a*-silenced larvae. Postembryonic root damage was lower and no longer different from damage to embryonic roots for *bx1* mutants infested with *DvvGr43a*-silenced larvae (Fig 4D). Thus, both benzoxazinoids and sugars increase root damage in an additive manner, and this pattern is driven by postembryonic root damage. Nevertheless, bite marks were still observed on 69% of *bx1* mutant roots infested with *DvvGr43a*-silenced larvae, showing that the larvae attack maize roots even in the absence of these cues. Taken together, these results provide evidence for distinct and additive roles of benzoxazinoids and sugars in root and tissue location and feeding preferences of the western corn rootworm.

### Using benzoxazinoids and sugars as foraging cues improves herbivore growth and survival

Herbivore foraging theory predicts that herbivores use plant chemical cues to make fitness-relevant foraging decisions. To test whether the integration of primary and secondary metabolites into foraging improves herbivore performance and survival, we first evaluated whether the absence of benzoxazinoids or the inability to detect sugars may have direct performance effects on the western corn rootworm (as opposed to the effects mediated by changes in foraging behavior). Benzoxazinoids improve western corn rootworm performance under iron-limiting conditions [72] and upon attack by entomopathogenic nematodes [85,86]. When bioavailable iron is abundant and natural enemies are absent, larval performance under no-choice conditions is not altered by benzoxazinoids [72,75]. Thus, growing maize plants with bioavailable EDTA-Fe as iron source allowed us to exclude direct effects of benzoxazinoids on larval performance. To test whether silencing *DvvGr43a* has direct negative effects on western corn rootworm performance, we reared control and *DvvGr43a*-silenced larvae on young maize seedlings that produce embryonic, but no postembryonic roots. In this way, larvae were restricted to feed on one root type without the need of experimental removal of roots or other interventions that could have biased the experiment. Larval weight gain and mortality of control and *DvvGr43a-*silenced larvae after 6 days was similar (S8 Fig), demonstrating that silencing *DvvGr43a* has no direct effects on larval performance in a no-choice situation. Note that larval mortality in this experiment was >40%, which is within the normal range for early instars of this species [87,88].

**Fig 5:**
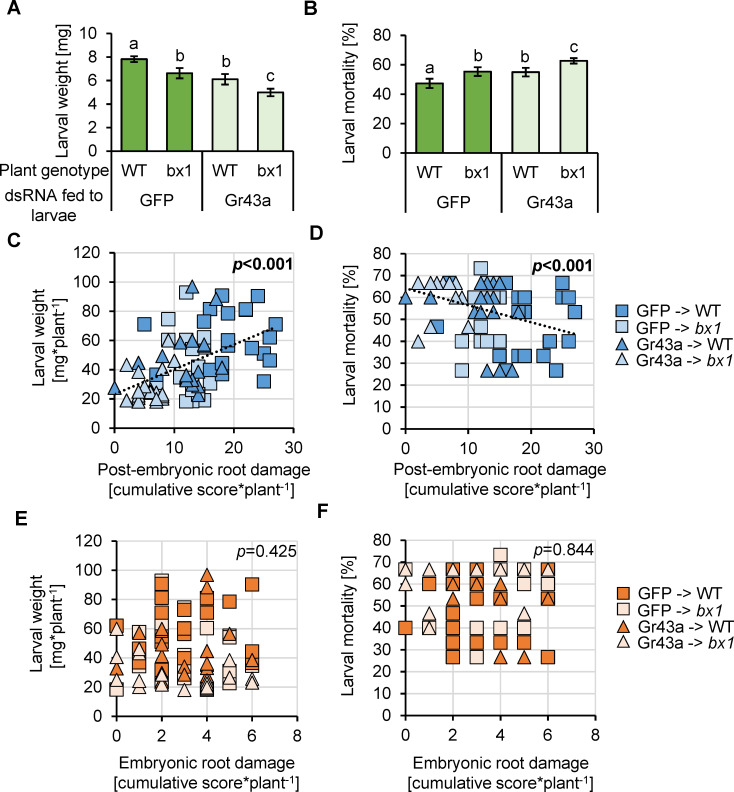
Using benzoxazinoids and sugars as foraging cues improves herbivore growth and survival. (A) Weight of control (GFP) and *DvvGr43a*-silenced larvae feeding on WT B73 or *bx1* mutant plants for 7 days. Note that in a no-choice situation, neither the *bx1* mutation nor *DvvGr43a* silencing reduce larval performance ([75] and S8 Fig). Letters indicate significant differences (*p* < 0.05, Holm–Sidak post hoc tests, *n* = 20 pots with 15 larvae each). (B) Larval mortality within the same experiment. Letters indicate significant differences (*p* < 0.05, Holm–Sidak post hoc tests, *n* = 20 pots with 15 larvae each). (C–F) Correlations between cumulative damage per plant and larval performance parameters for postembryonic roots (C, D) and embryonic roots (E, F). Linear regressions are shown for significant correlations (*p* < 0.05). *P* values are shown for Spearman Rank Order correlations. Underlying data can be found in S1 Data. dsRNA, double-stranded RNA; GFP, green fluorescent protein; WT, wild-type.

Having established the validity of our experimental approach, we proceeded to evaluate the impact of benzoxazinoid- and sugar-dependent foraging behavior on western corn rootworm performance. Highest larval weight and lowest mortality was observed for control larvae feeding on WT plants (Fig 5A and 5B). Intermediate weight gain and mortality was observed for control larvae feeding on *bx1* mutant roots and for *DvvGr43a*-silenced larvae feeding on WT roots. Lowest weight gain and highest mortality was observed for *DvvGr43a*-silenced larvae feeding on *bx1* mutant roots. Larval performance thus mirrored root damage patterns (Fig 4C and 4D). This was confirmed by correlation analysis which revealed a positive relationship between total larval weight and survival on a given plant and the total damage observed on postembryonic roots (Fig 5C–5F). In conclusion, we infer that sugars and benzoxazinoids act together to improve western corn rootworm performance by acting as foraging cues that guide the herbivore to feed on the most profitable host tissue.

## Discussion

Plant primary and secondary metabolites can act together to determine the behavior of generalist herbivores on artificial diet [33,34]. The present work expands these findings by providing evidence for distinct, overlapping, and additive roles of primary and secondary metabolites as determinants of the behavior, and, consequently, the performance of a specialist herbivore in planta.

Herbivores are thought to use primary and secondary metabolites to make foraging decisions within complex host metabolomes [16], but how they combine these 2 types of cues during different stages of host finding and acceptance is not well understood. From the experiments presented here, we infer that the western corn rootworm employs sugars for overall host finding. Sugars also increase the successful localization of postembryonic roots, but only in the presence of benzoxazinoids, which are essential for the latter. In contrast to these distinct roles, sugars and benzoxazinoids act in an additive manner in enhancing overall root damage and tissue-specific postembryonic root damage. Thus, western corn rootworm larvae employ primary and secondary metabolites in varying combinations and hierarchies for different behavioral patterns. This finding differs from current hypotheses and observations for polyphagous insects that suggest sequential use of different foraging cues [89,90]. The explanation for this discrepancy is likely found in the distribution patterns of plant primary and secondary metabolites in maize roots and rhizosphere. Benzoxazinoids and sugars constantly accumulate in maize roots, with stable concentration differences between postembryonic and embryonic roots [72,75]. Both compound classes are also exuded into the rhizosphere [91,92]. Current work using enzyme-inhibiting and noninhibiting extraction solvents [72,75,91] suggests that benzoxazinoids are likely exuded as glucosides before being deglycosylated at the root surface, resulting in the release of aglucones and glucose. Aglucones such as DIMBOA then form attractive complexes with free iron [72]. Thus, both sugars and benzoxazinoids are present inside and outside of the roots, with outside accumulation likely being more dynamic than inside accumulation. Taking into account that benzoxazinoids are plant-specific, while sugars are more general cues [93,94], one can envisage a scenario where the western corn rootworm, after having located a host plant through long-distance volatile cues [73], relies on sugars as general short-distance cues for the presence of roots (with potentially high concentrations on the surface of maize roots due to benzoxazinoid deglycosylation), followed by benzoxazinoids as specific within-plant tissue selection cues. Benzoxazinoids may also be used as specific short-distance host recognition cues in case roots of multiple plant species are intermingled. Following host and tissue finding, the herbivore would then use both sugars and benzoxazinoids as stable host acceptance and feeding cues. High-resolution metabolite imaging [95] together with the manipulation of GRs [23] could help to test these hypotheses and explore the connections between the distribution dynamics of different plant metabolites and herbivore foraging.

Plants produce complex chemical mixtures, and herbivores likely combine many of these chemicals to make appropriate foraging decisions [11,12,29]. In line with this idea, recent in vivo work shows that, while individual compound classes and insect GRs can be of major importance for specific behavioral patterns, their disruption is not sufficient to fully suppress these behaviors [22–24]. In the case of the western corn rootworm, we find that even the suppression of 2 major classes of foraging cues does not fully abolish its ability to find and accept a host plant, at least under laboratory conditions. Several other plant-derived chemicals, including different volatiles and fatty acids, are known to attract, arrest, and stimulate feeding by the western corn rootworm [67,68,70]. These cues are likely to enable basic behavioral patterns in the absence of sugars and benzoxazinoids. Whether the residual levels of benzoxazinoids that are still present in *bx1* mutant roots contribute to foraging success remains to be determined. *bx1*.*igl* double mutants that no longer produce benzoxazinoids are only available in a segregating genetic backgrounds, thus making direct comparisons difficult [86]. However, under no choice-conditions, western corn rootworm larvae have been observed to attempt to feed on rice, a plant species that does not produce benzoxazinoids [72], thus demonstrating that some degree of host acceptance also occurs in the absence of these chemicals. We conclude that the flexible integration of complex blends of chemical cues provides partial resistance to chemically mediated behavioral disruption. Recent work highlights the importance of learning in herbivore foraging [12,96], which likely increases the capacity of herbivores to respond to dynamic chemical landscapes and to substitute between different cues with redundant information content. How previous experiences influence the foraging behavior of the western corn rootworm remains to be determined. All larvae used in our experiments were reared on maize, reflecting their high degree of specialization on this host plant [65]. Western corn rootworm adults are more flexible with their diet [65,97], and it would be interesting to understand how their experience influences the responses of the next larval generation to host plant cues.

The capacity of an herbivore to select a host plant and feed on specific tissues generally improves its fitness [40,42,98]. In line with these preference–performance relationships, diet experiments often document strong associations between chemically mediated herbivore feeding preferences and herbivore performance [17]. In many of these cases, herbivore feeding is directly elicited by substances that improve performance [17]. However, plant metabolomes are likely too complex to be fully assessed by an herbivore, who thus need to rely on digestive feedbacks or subsets of chemical cues to make decisions. To what extent the use of plant chemical cues enhances herbivore performance by guiding herbivores to nutritious tissues is poorly understood, and interactions between primary and secondary metabolite chemical cues have, to the best of our knowledge, not been tested in planta. Our experimental approach rules out direct effects of benzoxazinoids and sugar perception on western corn rootworm performance [72,75] and thus allows us to quantify their net impact as foraging cues. We find that the availability of sugars and benzoxazinoids during foraging improves herbivore weight gain and survival individually by 8%, and in combination by 15%, benefits which are most likely driven by increased postembryonic root feeding. Postembryonic roots of young maize plants are growing more vigorously than embryonic roots, receive a higher share of photosynthates and are richer in carbohydrates, amino acids, and total soluble protein, and increase western corn rootworm growth in no-choice experiments [75]. In combination, these findings demonstrate that the combined use of primary and secondary metabolites as foraging cues enhance the performance of a specialist insect herbivore by guiding it to its most profitable feeding niche.

The gustatory receptor Gr43a is a highly conserved insect sugar receptor that is narrowly tuned to D-fructose [49] but mediates feeding preferences for different sugars through dietary feedbacks [55]. Given the conserved function, predicted protein structure, tissue-specific expression pattern, and specific effect on sugar preferences, *DvvGr43a* is very likely to act as a sugar sensor in the western corn rootworm as well. An interesting observation in this context is that *DvvGr43a*-dependent larval feeding preferences for sugars and sugar mixtures are only established after 2 to 3 hours, while feeding preferences for Fe(III)(DIMBOA)_3_ are established more rapidly [72]. In Drosophila, Gr43a increases sugar intake under food limiting conditions and suppresses sugar intake under satiation [49]. We thus hypothesized that the delayed *DvvGr43a*-dependent sugar feeding preference of western corn rootworm larvae for sugars may be the result of a starvation response. However, we observed a similar lag time in sugar preference in prestarved larvae. This suggests that *DvvGr43a* likely functions as a sugar sensor rather than as a nutrient uptake regulator in the western corn rootworm. The observed lag phase may be explained by the potential role of *DvvGr43a* as an internal sugar sensor that triggers behavioral responses once sugars are taken up and elicit changes in hemolymph sugar levels. A deeper molecular characterization could shed light on the specificity and precise neurological and physiological roles of *DvvGr43a* in the western corn rootworm in the future.

The western corn rootworm is an important and damaging maize pest, and a better understanding of its chemical ecology may help to guide efforts toward new control approaches [65]. To fully appreciate the impact of different maize chemicals on herbivore foraging and pest status, additional experiments in the field to assess the role of different feeding cues in determining herbivore fitness and plant yield would be important. Nevertheless, the present work, together with previous work on the chemical ecology of the western corn rootworm [65,72,74,86], illustrates how tightly this insect is associated with the chemistry of its host plant. Diluting the availability of maize through multiyear crop rotation thus represents an excellent and indeed established strategy to control this pest [99]. Undersowing with other plant species was not found to be efficient [100], most likely because of the robustness of the host finding capacity of the western corn rootworm larvae in the soil. Biotechnological approaches could also be used to introduce nonhost plant chemistry into maize roots in order to confuse or even kill the western corn rootworm [101]. As the western corn rootworm does not develop well on roots of older maize plants [102], which likely also produce different sets of root secondary metabolites [61,103], approaches to express these chemicals in younger plants could also be a promising avenue in addition to existing transgenic strategies based on RNAi and *Bacillus thuringiensis* (*Bt*) toxins [104]. However, given the robustness of the foraging behavior of the western corn rootworm and its capacity to adapt to new defenses such as *Bt*-toxins [105], such approaches may be short-lived if not accompanied by robust resistance management.

In conclusion, the experiments presented here reveal how primary and secondary metabolites interact to guide the foraging of a specialist herbivore and important root pest. The distinct individual and combined roles of these metabolites reveal finely tuned herbivore foraging strategies that are directly linked to improved herbivore performance and survival. At the same time, foraging is remarkably robust to perturbation, most likely due to the flexible use of redundant host plant cues. Our mechanistic approach thus offers insights into the complex, multilayered interplay between plant metabolomes and chemically guided herbivore foraging.

## Materials and methods

### Plants and insects

Maize seeds (*Zea mays* L., inbred line B73) were provided by Delley Semences et Plantes SA (Delley, CH). The near-isogenic benzoxazinoid deficient *bx1* mutant line in a B73 background was obtained by backcrossing the original *bx1* mutant 5 times into B73 [106]. Seedlings were grown under greenhouse conditions (23 ± 2°C, 60% relative humidity, 16:8 h L/D, and 250 mmol/m^2^/s^1^ additional light supplied by sodium lamps). Plantaaktiv 16+6+26 Typ K fertilizer (Hauert HBG Dünger AG, Grossaffoltern, Switzerland) was added twice a week after plant emergence following the manufacturer’s recommendations. When plants were used to feed insects, seedlings were germinated in vermiculite (particle size: 2 to 4 mm; tabaksamen, Switzerland) and used within 4 days after germination. Eggs of the nondiapausing *Diabrotica virgifera virgifera* strain were originally supplied by USDA-ARS-NCARL, Brookings, South Dakota. Insect colonies were subsequently established and are maintained in the University of Bern and the University of Neuchatel. Upon egg hatching, insects were maintained in organic soil (Selmaterra, Bigler Samen AG, Thun, Switzerland) and fed freshly germinated maize seedlings.

### Herbivore behavior

To evaluate the preference of western corn rootworm larvae for different plant genotypes and root types, we use 3 different experimental setups: potted plants, root systems laid out in petri dishes, and detached roots in petri dishes.

For western corn rootworm feeding preference experiments using whole plants, 3-week-old maize plants grown in 200 mL cylindrical plastic pots filled with sand and topped off with potting soil were infested with 15-second instar western corn rootworm larvae. Twenty plants per plant genotype/type of larvae combination were evaluated (*n* = 20). Seven days after infestation, plants were gently excavated and inspected for root damage. Damage was recorded for each root individually using the following scale. 0: no visual damage, 1: one-three bite marks, 2: more than 3 bite marks, 3: several bite marks and brown discoloration, 4: root partially removed, and 5: root fully removed.

Western corn rootworm feeding preference assays using petri dishes were conducted as described [107,108]. Briefly, 2-week-old maize plants grown as described above were gently excavated. The root systems were briefly rinsed with water and laid out onto a moist filter paper embedded in a petri dish (14 cm diameter, Greiner Bio-one, Austria). A circular whole in the rim of the dishes accommodated the plant stems, with the shoots remaining outside of the dishes. Five third instar western corn rootworm larvae were released into each petri dish. Petri dishes were sealed and then covered with aluminum foil. The number of western corn rootworm larvae feeding on each root type was determined at different time points after their release.

To evaluate western corn rootworm feeding preference using detached roots, pieces of comparable length and diameter of 2 postembryonic roots and 2 embryonic roots from 2-week-old maize plants were placed next to each other onto a moist filter paper (diameter 90 mm, GE Healthcare, United Kingdom) embedded into a petri plate (90 mm diameter, Greiner Bio-one, Austria). Five third instar western corn rootworm larvae were released into each petri dish. Petri plates were sealed and then covered with aluminum foil. The number of western corn rootworm larvae feeding on each root type was determined at different time points.

To evaluate the attractiveness of CO_2_ for western corn rootworm larvae, we conducted choice experiments using belowground olfactometers [70]. CO_2_ levels were increased in one arm of the olfactometer by delivering CO_2_-enriched synthetic air (1% CO_2_, Carbagas, Switzerland). Olfactometer arms were closed on top using parafilm during CO_2_ delivery. A manometer connected to the synthetic air bottle allowed to fine-tune CO_2_ delivery rates. CO_2_ levels were measured using a gas analyzer (Li7000, Li-Cor, Lincoln, Nebraska, United States of America). Once desired CO_2_ concentrations were reached, CO_2_ delivery was terminated, olfactometer arms were connected to olfactometer central connectors and 6 seconds to third instar western corn rootworm larvae were release immediately, and their choice were evaluated within 10 minutes of release. Six olfactometers per larval type were assayed (*n* = 6).

To evaluate the attractive effects of Fe(III)(DIMBOA)_3_ for western corn rootworm larvae, we release 6 seconds to third instar larvae in the middle of a moist sand-filled petri dish (9 cm diameter, Greiner Bio-One GmbH, Frickenhausen, DE) where they encountered a filter paper disc treated with 10 μl of Fe(III)(DIMBOA)_3_ (1 μg*ml^−1^ of water) or, on the opposite side, a filter paper disc treated with water only. Ten independent petri dishes per larval type were evaluated (*n* = 10). Fe(III)(DIMBOA)_3_ was prepared fresh by mixing FeCl_3_ and DIMBOA at a 1:2 ratio as described [108]. Larval preferences were recorded 1 hour after releasing the larvae.

To evaluate the capacity of Fe(III)(DIMBOA)_3_ to complement the *bx1* mutant phenotype, assays with 3-week-old WT and *bx1* mutant maize plants were conducted as described in the section “western corn rootworm feeding preference experiments using whole plants.” Two hundred μl of a Fe(III)(DIMBOA)_3_ solution (50 μg*ml^−1^ of water) were applied to the postembryonic roots of a subset of the *bx1* plants. Postembryonic roots of WT plants and of an additional subset of *bx1* plants received water only. One hour later, 6 third instar western corn rootworm larvae were released into each petri dish. Petri dishes were sealed and then covered with aluminum foil. The number of western corn rootworm larvae feeding on each root type was determined at different time points after their release in 10 different petri dishes per treatment (*n* = 10).

To evaluate attractive effects of soluble sugars for western corn rootworm larvae, we followed the procedure described by Bernklau and colleagues with minor modifications [109]. Briefly, we released 6 seconds to third instar western corn rootworm larvae in the middle of a moist sand-filled petri dish (6 cm diameter, Greiner Bio-One GmbH, Frickenhausen, DE) where they encountered a filter paper disc treated with 10 μl of either glucose (30 mg*ml^−1^), fructose (30 mg*ml^−1^), sucrose (30 mg*ml^−1^), or a mixture of the three (10 μl containing each sugar at a concentration of 30 mg*ml^−1^), or, on the opposite side, a filter paper treated with water only. Fifteen independent petri dishes per treatment were assayed (*n* = 15). Larval preferences were recorded regularly for 3 hours. In a second experiment, larvae were pre-fed on maize roots or starved for 12 hours before being released into petri dishes with filter paper discs treated with a sugar mixture or water, as described above. Eight independent petri dishes with 6 larvae each were assayed (*n* = 8). In a third experiment, larval responses to sugar mixtures at different concentrations of individual sugars (3 to 100 mg*ml^−1^) were assayed as described above. Five independent petri dishes with 6 larvae each were assayed (*n* = 5).

### Herbivore performance

To evaluate the impact of benzoxazinoids and sugars on western corn rootworm performance, 3-week-old maize plants grown in 200 mL cylindric plastic pots (11 cm depth and 4 cm diameter) filled with sand were infested with 15-second instar western corn rootworm larvae (*n* = 20). Seven days after infestation, the larvae were collected, counted, and weighted using a microbalance. Note that weight and survival was determined on the same plants as root damage (see section “Herbivore behavior”).

To evaluate the impact of *DvvGr43a* silencing on western corn rootworm performance in a no-choice setting, GFP and *DvvGr43a* fed first instar larvae were reared on 3- to 4-day-old maize seedlings, which only produce embryonic roots, in soil-filled plastic cups (*n* = 40). Nine larvae were used per cup. Fresh seedlings were provided to the larvae every 3 days for a total of 6 days, after which the larvae were collected, counted, and weighted using a microbalance.

### Root metabolite measurements

Untargeted root metabolomics was conducted as described [86]. Embryonic and postembryonic roots of B73 plants were harvested separately, flash-frozen, and ground in liquid nitrogen. Sixty to 80 mg of plant tissues were then extracted in 800 μL of acidified H_2_O/MeOH (50:50 v/v; 0.1% formic acid). Resulting root extracts were analyzed using an Acquity UHPLC system coupled to a G2-XS QTOF mass spectrometer equipped with an electrospray source (Waters, Milford, Massachusetts, USA). Gradient elution was performed on an Acquity BEH C18 column (2.1 × 50 mm i.d., 1.7 μm particle size) at 1% to 27.5% B over 3.5 minutes, 27.5% to 100% B over 1 minute, holding at 100% B for 1 minute, and reconditioning at 1% B for 1 minute, where A = 0.1% formic acid/water and B = 0.1% formic acid/acetonitrile. The flow rate was 0.4 mL*min^−1^. The temperature of the column was maintained at 40°C, and the injection volume was 1 μL. The QTOF MS was operated in negative mode. The data were acquired over an m/z range of 100 to 1,200 with scans of 0.15 second at collision energy of 4 V and 0.2 second with a collision energy ramp from 10 to 40 V. The capillary and cone voltages were set to 2 kV and 20 V, respectively. The source temperature was maintained at 140°C, the desolvation gas was 400°C at 1,000 L h^−1^, and cone gas flow was 50 L*h^−1^. Accurate mass measurements (<2 ppm) were obtained by infusing a solution of leucin encephalin at 200 ng*ml^−1^ at a flow rate of 10 μL*min^−1^ through the Lock Spray probe (Waters). The chromatograms were processed using Progenesis QI (Nonlinear Dynamics, Newcastle, UK) with default settings for spectral alignment, peak picking, and deconvolution, resulting in a total of 4,956 mass features. Of these, 444 mass features eluting between 0 and 0.3 minutes were discarded, since their lack of retention and excessive coelution made reliable identification impossible. In addition, masses with decimals >0.8 were excluded, as such masses do not correspond to feasible compounds of biological origin and are likely instrument artifacts. Finally, mass features that eluted within a window of 0.04 minutes of each other and were correlated with r > 0.8 (Pearson correlation) across all samples were considered fragments of the same compound and grouped together, selecting one mass feature per feature group. If a feature group contained an identified compound, its [M−H]− mass feature was retained; alternatively, the most abundant mass feature was retained in each feature group.

Abundances for each remaining mass feature were normalized by ArcSinH transformation, and mean normalized abundances for postembryonic and embryonic WT roots were compared by Student *t* test. *P* values were adjusted by Benjamini–Hochberg false discovery rate (FDR) multiple testing correction and a fold-change cut-off of 2.0 was applied to generate a list of compounds that significantly differed between treatments, and volcano plots were used to visualize treatment differences. The list of differentially expressed features was tested against an in-house mass fragmentation database using Progenesis QI (Progenesis MetaScope v1.0.6253.26544, Nonlinear Dynamics, Newcastle, UK), and chromatograms were manually scanned for exact masses of known plant metabolites, specifically amino acids, sugars, organic acids, and flavonols. Putative identifications were accepted based on exact mass, mass fragmentation patterns, and relative retention times. To evaluate chemical similarity of WT and *bx1* mutant plants, we performed a two-way ANOVA for each ArcSinH-transformed mass feature, testing for main effects of root type, genotype, and an interaction effect. *P* values were again adjusted by Benjamini–Hochberg FDR multiple testing correction.

Root benzoxazinoid quantification was conducted as described [110]. For this, embryonic and postembryonic roots of B73 or *bx1* plants were harvested separately, flash-frozen, and ground in liquid nitrogen. Approximately 100 mg of plant tissues were then extracted in 1 mL of acidified H_2_O/MeOH (50:50 v/v; 0.1% formic acid), and analyzed with an Acquity UHPLC system coupled to a UV detector and a Waters QDa mass spectrometer equipped with an electrospray source (Waters). Compounds were separated on an Acquity BEH C18 column (2.1 × 50 mm i.d., 1.7 μm particle size). Water (0.1% formic acid) and acetonitrile (0.1% formic acid) were employed as mobile phases A and B. The elution profile was: 0 to 1 minute, 10% B; 1 to 4 minutes, 10% to 30% B; 4 to 5 minutes, 30% to 9740% B; 5 to 6 minutes, 40% to 100% B; 6 to 8.5 minutes, 100% B; followed by 1.5 minute reconditioning at 10% B. The mobile phase flow rate was 0.4 mL*min^−1^. The column temperature was maintained at 40°C, and the injection volume was 3 μL. The MS was operated in negative mode and data were acquired in single ion recording (SIR) mode, using [M−H]^−^ and [M+HCOO^−^]^−^ adducts of benzoxazinoid compounds. All other MS parameters were left at their default values as suggested by the manufacturer. Benzoxazinoid peaks were identified from MS signals and quantified from integrated UV signals at 265 nm. External standards for HMBOA, DIMBOA, DIMBOA-Glc, HDMBOA-Glc, and MBOA were used for absolute quantification. Where concentrations were too low to result in reliable UV signals, a compound-specific MS-to-UV signal conversion factor was estimated to calculate “predicted” UV signals from the more sensitive MS signals before quantification.

Root sugar levels were measured by an enzymatic/spectrophotometric method as described [111]. Embryonic and postembryonic roots of B73 and *bx1* plants were harvested separately, flash-frozen, and ground in liquid nitrogen. Soluble sugars were extracted from 100 mg root tissue using 80% (v/v) ethanol, followed by an incubation step (10 minutes at 78°C) with constant shaking at 800 rpm. Pellets were reextracted twice with 50% (v/v) ethanol (10 minutes at 78°C with constant shaking at 800 rpm). Supernatants from all extraction steps were pooled together, and sucrose, glucose, and fructose were quantified as described [112].

### Identification of *DvvGr43a*

To identify the *Gr43a* receptor orthologues in the western corn rootworm, we used the *Gr43a* receptor-encoding gene sequence of *Drosophila melanogaster* as query against publicly available nucleotide sequences and against the western corn rootworm genome sequences (NCBI accession: PXJM00000000.2) using the National Center for Biotechnology Information Basic Local Alignment Search Tool (NCBI BLAST). The full gene sequence can be retrieved from the National Center for Biotechnology Information (NCBI) data bank using the following accession number: XM_028279548.1. Then, we reconstructed evolutionary relationships between the identified DvvGr43a gene and several other putative Gr43a genes that have been functionally characterized as glucose, fructose, and/or sucrose receptors [49,50,53,54,80–83]. The evolutionary relationships were inferred using the Maximum Likelihood method based on the Le Gascuel 2008 model in MEGA7 using protein sequences [113,114]. The tree with the highest log likelihood (−14287.71) is shown. The percentage of trees in which the associated taxa clustered together is shown next to the branches. Initial tree(s) for the heuristic search were obtained automatically by applying Neighbor-Join and BioNJ algorithms to a matrix of pairwise distances estimated using a JTT model, and then selecting the topology with superior log likelihood value. A discrete Gamma distribution was used to model evolutionary rate differences among sites (5 categories (+G, parameter = 1.0811)). The tree is drawn to scale, with branch lengths measured in the number of substitutions per site. There was a total of 569 positions in the final dataset. Graphical representation and edition of the phylogenetic tree were performed with the Interactive Tree of Life (version 3.5.1) [115]. Protein tertiary structures and topologies were predicted using Phyre2 [116]. The protein sequences used to reconstruct evolutionary relationships can be retrieved from the NCBI under the following accession numbers: *Heliothis virescens* Gr4 (CAD31946.1), *Danaus plexippus* Gr4 (EHJ77681.1), *Bombyx mori* Gr9 (NP_001124345.1), *Bombus impatiens* Gr43a (XP_003486787.1), *Nasonia vitripennis* Gr3 (NP_001164386.1), *Apis mellifera* Gr3 (XP_016768876.1), *Drosophila melanogaster* Gr43a (NP_523650.2), *Tribolium castaneum* Gr20 (EFA05758.1), *Anopheles gambiae* Gr43a (XP_318100.4), *Camponotus floridanus* Gr43a (EFN61344.1), *Aedes aegypti* Gr43a (XP_001658898.3), *Culex quinquefasciatus* Gr43a (XP_001842305.1), *Orussus abietinus* Gr43a (XP_012270798.1), *Aethina tumida* Gr43a (XP_019876958.1), *Anoplophora glabripennis* Gr43a (XP_018574194.1), *Helicoverpa armigera* Gr4 (AGK90011.1), *Helicoverpa armigera* Gr9 (JX970522.1), *Trichogramma chilonis* Gr43a (QAY30709.1), and *Diabrotica virgifera* Gr43a (XP_028135349.1) [117]. For *Plutella xylostella* GR43a-1 and *Plutella xylostella* GR43a-2 GRs refer to Liu and colleagues as these sequences have not been deposited in a public repository [53].

### Double-stranded RNA production

dsRNA targeting *DvvGr43a* was produced by *Escherichia coli* HT115. To this end, electrocompetent *E*. *coli* HT115 cells were transformed with recombinant L4440 plasmids that contained either a 240-bp long *DvvGr43a* gene fragment, or a 240-bp long GFP gene fragment flanked by 2 T7 promotors in opposite directions. Gene fragments were synthetized de novo (Eurofins, Germany). To induce the production of dsRNA, an overnight bacterial culture was used to inoculate fresh Luria-Berthani broth (25 g*L^−1^, Luria/Miller, Carl Roth GmbH, Karlsruhe, Germany). Once the bacterial culture reached an OD_600_ of 0.6 to 0.8, it was supplemented with isopropyl β-D-1-thiogalactopyranoside, IPTG) (Sigma-Aldrich, Switzerland) at a final concentration of 2 mM. Bacterial cultures were incubated at 37°C in an orbital shaker at 130 rpm for 16 hours. Bacteria were harvested by centrifugation and stored at −20°C for further use [118].

### Gene silencing experiments

To induce gene silencing in western corn rootworm, 6 to 10 western corn rootworm larvae were released in solo cups (30 mL, Frontier Scientific Services, Germany) containing approximately 2 g of autoclaved soil (Selmaterra, Bigler Samen AG, Thun, Switzerland) and 2 to 3 freshly germinated maize seedling. Maize seedlings were coated with 1 ml of bacterial solution containing approximately 200 to 500 ng of dsRNA targeting *DvvGr43a*. As controls, larvae were fed with bacteria producing dsRNA targeting GFP genes. Seedlings coated with bacteria were provided every other day for 3 consecutive times. Two days after, larvae were collected and used for experiments.

### Gene expression measurements

Total RNA was isolated from approximately 10 mg of frozen, ground, and homogenized western corn rootworm larval tissue (3 to 7 larvae per biological replicate, n = 8 to 11) using the GenElute Universal Total RNA Purification Kit (Sigma-Aldrich, St. Louis, Missouri, USA). A NanoDrop spectrophotometer (ND-1000, Thermo Fisher Scientific, Waltham, Massachusetts, USA) was used to estimate RNA purity and quantity. DNase-treated RNA was used as template for reverse transcription and first strand cDNA synthesis with PrimeScript Reverse Transcriptase (Takara Bio, Kusatsu, Japan). DNase treatment was carried out using the gDNA Eraser (Perfect Real Time) following manufacturer’s instructions (Takara Bio). For gene expression analysis, 2 μl of undiluted cDNA (i.e., the equivalent of 100 ng total RNA) served as template in a 20-μl qRT-PCR using the TB Green Premix Ex Taq II (Tli RNaseH Plus) kit (Takara Bio) and the Roche LightCycler 96 system (Roche, Basel, Switzerland), according to manufacturer’s instructions. Transcript abundance of the following western corn rootworm genes were analyzed: *DvvGr43a* and *DvvGr2*. *Actin* was used as reference gene to normalize expression data across samples. Relative gene expression levels were calculated by the 2^−ΔΔCt^ method [119]. The following primers were used: DvvGr43a-F GTCACATTCACCACGGGTCT, DvvGr43a-R CGTTCGGTCTCTAACTTTGGC, DvvGr2-F GAACTAAGCGAGCTCCTCCA, DvvGr2-R CAGAAGCACCATGCAATACG, DvvActin-F TCCAGGCTGTACTCTCCTTG, and DvvActin-R CAAGTCCAAACGAAGGATTG.

### Statistical analyses

Differences in root benzoxazinoid and root sugar levels as well as gene expression levels were analyzed by one- and two-way analyses of variance (ANOVAs). Normality and equality of variance were verified using Shapiro–Wilk and Levene tests, respectively. Holm–Sidak post hoc tests were used for multiple comparisons. Datasets from experiments that did not fulfill the assumptions for ANOVA were natural log-, root square-, or rank-transformed before analysis. Differences in damage scores between embryonic and postembryonic roots were assessed using Wilcoxon Signed Rank tests. Differences in average root damage scores between treatments were assessed using Kruskal–Wallis ANOVAs on ranks followed by Tukey post hoc tests. The above analyses were performed in Sigma Plot 12.0 (SystatSoftware, San Jose, California, USA) with the support of the built-in Advisor Wizard function. Differences in larval preference were assessed through Generalized Linear Models (GLM) with binomial distribution and corrected for overdispersion with quasi-binomial function when necessary followed by ANOVA and FDR-corrected Least Square Means post hoc tests. These analyses were followed by residual analysis to verify the suitability of the error distribution and model fitting. These Analyses were conducted using R 3.2.2 (43) using the packages “lme4,” “car,” “lsmeans,” and “RVAideMemoire” [120–124]. Mass features were normalized using the function *arcsinh_x* in the R package “bestNormalize” and compared between root types using functions *t*.*test* in base R and *foldchange* in the package “gtools.” *P* values were FDR-corrected using the function *p*.*adjust*.

## Supporting information

S1 Data Data generated for this manuscript. (XLSX)

S1 Table Identified metabolites in maize postembryonic and embryonic roots. Metabolites with retention times highlighted by asterisks (*) eluted together with many weakly retained compounds that could not be separated by reversed-phase liquid chromatography (RP-UHPLC). Their identification is therefore tentative and based on accurate masses only.
(PDF)

S2 Table Root metabolites with behavioral activity toward western corn rootworm larvae. (PDF)

S1 Fig Frequency of different types of damage on individual embryonic and postembryonic roots. Data are from experiment shown in Fig 1B (*n* = 20 plants). E1–E2 refer to individual embryonic (E) roots, PE1–PE6 refer to individual postembryonic (PE) roots. Numbers were assigned randomly to embryonic and postembryonic roots within plants. The sizes of the circles are proportional to the relative frequency (% within each root) of the different types of observed damage. Underlying data can be found in S1 Data.
(PDF)

S2 Fig Metabolic differences between embryonic and postembryonic roots are conserved in the bx1 mutant. Relative abundances (signal intensities) of identified metabolic features in embryonic and postembryonic roots of WT B73 and *bx1* mutant plants (*n* = 7–10). For sugars and benzoxazinoids, refer to Fig 2. Results of two-way ANOVAs for genotype effects (G), root type effects (R), and their interaction (GxR) are shown for each compound (***p < 0.001, **p < 0.01, *p < 0.05). Error bars denote standard errors of means (SEM). Underlying data can be found in S1 Data.
(PDF)

S3 Fig Residual concentrations of benzoxazinoids in the bx1 mutant do not differ between root types. Concentrations of benzoxazinoids in embryonic and postembryonic roots of *bx1* mutant plants (*n* = 11–18). Figure is an expanded panel of Fig 2. No significant differences were found for individual compounds (*p* > 0.05, Holm–Sidak post hoc tests following two-way ANOVAs). Error bars denote standard errors of means (SEM). Underlying data can be found in S1 Data.
(PDF)

S4 Fig Exogenous Fe(III)(DIMBOA)_3_ is sufficient to restore postembryonic root preference of the western corn rootworm. Preference of WT western corn rootworm larvae for embryonic and postembryonic roots of wild type (WT) and *bx1* mutant plants in a petri dish assay. For a subset of *bx1* mutant root systems, the postembryonic roots were complemented with 200 μl of a Fe(III)(DIMBOA)_3_ solution at a concentration of 50 μg*ml^−1^ (v/v) in H_2_O. Embryonic roots were treated with an equal amount of H_2_O. Larval choice was recorded after 1 and 3 hours. Asterisks indicate significant preference (***p < 0.001, **p < 0.01, *p < 0.05, FDR-corrected Least Square Mean post hoc tests, *n* = 10 dishes with 6 larvae each). Underlying data can be found in S1 Data.
(PDF)

S5 Fig *DvvGr43a* does not influence the attraction of the western corn rootworm to CO_2_. Proportion of control (GFP) or *DvvGr43a*-silenced (Gr43a) larvae found on each arm of belowground olfactometers. Asterisks indicate significant differences between treatments (*p < 0.05, FDR-corrected Least Square Mean post hoc tests, 6 two-arm olfactometers with 6 larvae each were evaluated, *n* = 6). Underlying data can be found in S1 Data.
(PDF)

S6 Fig *DvvGr43a* mediates sugar preference independently of the feeding state of the western corn rootworm. Preference of satiated (left) and starved (right) control or *DvvGr43a*-silenced larvae for buffer or a glucose, fructose, sucrose mixture on filter discs at different time points (*p < 0.05, FDR-corrected Least Square Mean post hoc tests, 8 petri plates with 6 larvae each were assayed, *n* = 8). Error bars denote standard errors of means (SEM). Underlying data can be found in S1 Data.
(PDF)

S7 Fig *DvvGr43a* silencing disrupts sugar preferences at physiologically relevant concentrations. Preference of *GFP* or *DvvGr43a* dsRNA fed larvae for glucose, fructose, sucrose mixtures at different concentrations in H_2_O (v/v) on filter discs 3 hours after the start of the choice experiment. A volume of 10 μl of each individual sugar solution at the indicated concentration was added to the filter discs. Control filter discs were supplied with equal amounts of H_2_O (*p < 0.05; **p < 0.01; FDR-corrected Least Square Mean post hoc tests, *n* = 5 dishes with 6 larvae each). Error bars denote standard errors of means (SEM). Underlying data can be found in S1 Data.
(PDF)

S8 Fig *DvvGr43a* does not directly impair larval performance. (A) Weight of western corn rootworm larvae fed on GFP and DvvGr43a dsRNA on young maize seedlings that produce embryonic roots only (no-choice setting) for 7 days (*n* = 40 cups with 9 larvae each). (B) Larval mortality within the same experiment. Underlying data can be found in S1 Data.
(PDF)

## Abbreviations

dsRNA: double-stranded RNA
E: embryonic
FDR: false discovery rate
GFP: green fluorescent protein
GR: gustatory receptor
PE: postembryonic
RNAi: RNA interference
SEM: standard errors of means
WT: wild-type
