# Supplementary material for: The plant metabolome guides fitness-relevant foraging decisions of a specialist herbivore
Source: PLoS Biol. 2021 Feb 18;19(2):e3001114. doi: 10.1371/journal.pbio.3001114 (PMC7924754; doi:10.1371/journal.pbio.3001114)
Supplement: S1 Table — Metabolites with retention times highlighted by asterisks (*) eluted together with many weakly retained compounds that could not be separated by reversed-phase liquid chromatography (RP-UHPLC). Their identification is therefore tentative and based on accurate masses only. (PDF) [file pbio.3001114.s002.pdf]

Nr	Compound class	Compound Name	Molecular formula	Retention time [min]	MS adducts	Progenesis QI fragmentation score	Characteristic fragments [m/z]
1	Benzoxazinoid	DHBOA-Glc	$C_{14}H_{17}NO_9$	1.3	M-H, M+FA-H, 2M-H	16.5	180.0303, 162.0200
2	Benzoxazinoid	DIBOA-Glc	$C_{14}H_{17}NO_9$	1.91	M-H, M+FA-H, 2M-H	2.6	180.0303, 162.0200, 134.0247
3	Benzoxazinoid	HMBOA-Glc	$C_{15}H_{19}NO_9$	2.11	M-H, M+FA-H, 2M-H	10.3	194.0459, 166.0511
4	Benzoxazinoid	DIMBOA-Glc	$C_{15}H_{19}NO_{10}$	2.18	M-H, M+FA-H, 2M-H	5.9	210.0406, 192.0302, 164.0356, 149.0119
5	Benzoxazinoid	DIM$_2$BOA-Glc	$C_{16}H_{21}NO_{11}$	2.22	M-H, M+FA-H, 2M-H	21.7	222.0408, 194.0458
6	Benzoxazinoid	HMBOA	$C_9H_9NO_4$	2.3	M-H	n/a	-
7	Benzoxazinoid	DIMBOA	$C_9H_9NO_5$	2.42	M-H	n/a	164.0353, 149.0118
8	Benzoxazinoid	HDMBOA-Glc	$C_{16}H_{21}NO_{10}$	2.74	M-H, M+FA-H, 2M-H	11.5	224.0564, 194.0460
9	Benzoxazinoid	HDM$_2$BOA-Glc	$C_{17}H_{23}NO_{11}$	2.74	M+FA-H	n/a	254.0789
10	Organic acid	Chlorogenic acid	$C_{16}H_{18}O_9$	1.77	M-H, 2M-H	24.3	191.0562
11	Organic acid	Feruloylglucose	$C_{16}H_{20}O_9$	2.05	M-H	81	175.0404
12	Organic acid	Sinapoylglucose	$C_{17}H_{22}O_{10}$	2.09	M-H	36.7	223.0615, 205.0507
13	Organic acid	Quinic acid	$C_{17}H_{12}O_6$	0.33*	M-H, 2M-H	70.1	-
14	Organic acid	Coumaroylquinic acid isomer	$C_{16}H_{18}O_8$	1.7	M-H	98	163.0409
15	Organic acid	Coumaroylquinic acid isomer	$C_{16}H_{18}O_8$	2.13	M-H	51.6	191.0561, 173.0455
16	Organic acid	Feruloylquinic acid isomer	$C_{17}H_{20}O_9$	2.35	M-H	46.1	191.0562
17	Organic acid	Feruloylquinic acid isomer	$C_{17}H_{20}O_9$	2.61	M-H	n/a	-
18	Flavonol	Quercetin rhamnosylglucoside	$C_{27}H_{30}O_{16}$	2.66	M-H	65.7	301.0353
19	Flavonol	Quercetin glucoside	$C_{21}H_{20}O_{12}$	2.75	M-H	26	301.0347, 271.0252
20	Flavonol	Quercetin malonylglucoside	$C_{24}H_{22}O_{15}$	2.91	M-H	75.1	505.0986, 301.0345
21	Flavonol	Kaempferol rhamnosylglucoside	$C_{27}H_{30}O_{15}$	2.94	M-H	98.9	285.0412
22	Flavonol	Kaempferol glucoside	$C_{21}H_{20}O_{11}$	3.04	M-H	88.6	285.0395, 255.0304
23	Flavonol	Kaempferol malonylglucoside	$C_{24}H_{22}O_{14}$	3.25	M-H	96.5	489.1037, 285.0400
24	Amino acid	Leucine	$C_6H_{13}NO_2$	0.8	M-H	n/a	-
25	Amino acid	Phenylalanine	$C_9H_{11}NO_2$	1.1	M-H	n/a	-
26	Amino acid	Tryptophan	$C_{11}H_{12}N_2O_2$	1.5	M-H, M+FA-H	n/a	-
27	Sugar	Monosaccharide	$C_6H_{12}O_6$	0.31*	M-H	n/a	-
28	Sugar	Disaccharide	$C_{12}H_{22}O_{11}$	0.34*	M-H, M+FA-H, 2M-H	3.2	-
