# Supplementary material for: The plant metabolome guides fitness-relevant foraging decisions of a specialist herbivore
Source: PLoS Biol. 2021 Feb 18;19(2):e3001114. doi: 10.1371/journal.pbio.3001114 (PMC7924754; doi:10.1371/journal.pbio.3001114)
Supplement: S2 Table — (PDF) [file pbio.3001114.s003.pdf]

Nr	Compound class	Compound Name	Behavioral effect	Reference
1	Benzoxazinoid	MBOA	Host location (long-distance)	[1]
2	Benzoxazinoid	Fe-DIMBOA	Host location & acceptance (short-distance)	[2]
3	Benzoxazinoid	Fe-DIMBOA-Glc	Host location & acceptance (short-distance)	[2]
4	Sugar	Glucose	Feeding stimulation	[3]
5	Sugar	Fructose	Feeding stimulation	[3]
6	Sugar	Sucrose	Feeding stimulation	[3]
7	Fatty acid	Linoleic acid	Arrestment, feeding stimulation (within sugar blend)	[3]
8	Fatty acid	Oleic acid	Feeding stimulation (within sugar blend)	[3]
9	Fatty acid	Monoga-lactosyldiacylglycerol	Host recognition (tight turning behavior)	[4]
10	Aminobenzole	Methyl anthranilate	Host avoidance (repellent)	[5]
11	Terpene	(*E*)-β-caryophyllene	Host location and discrimination (long distance)	[6,7]
12	Alkene	Ethylene	Host location and discrimination (long-distance)	[8]
13	Inorganic compound	CO_2	Host location (long-distance)	[9]

REFERENCE

1. Bjostad LB, Hibbard BE. 6-Methoxy-2-benzoxazolinone: A semiochemical for host location by western corn rootworm larvae. J Chem Ecol. 1992; 18:931–44. doi: 10.1007/BF00980054 PMID: 24254139.
2. Hu L, Mateo P, Ye M, Zhang X, Berset JD, Handrick V, et al. Plant iron acquisition strategy exploited by an insect herbivore. Science. 2018; 361:694–7. doi: 10.1126/science.aat4082 PMID: 30115808
3. Bernklau EJ, Bjostad LB. Identification of feeding stimulants in corn roots for western corn rootworm (Coleoptera: Chrysomelidae) larvae. J Econ Entomol. 2008; 101:341–51. doi: 10.1093/jee/101.2.341.
4. Bernklau EJ, Hibbard BE, Dick DL, Rithner CD, Bjostad LB. Monogalactosyldiacylglycerols as host recognition cues for western corn rootworm larvae (Coleoptera: Chrysomelidae). J Econ Entomol. 2015; 108:539–48. doi: 10.1093/jee/tov025 PMID: 26470164.
5. Bernklau EJ, Hibbard BE, Norton AP, Bjostad LB. Methyl anthranilate as a repellent for western corn rootworm larvae (Coleoptera: Chrysomelidae). J Econ Entomol. 2016; 109:1683–90. doi: 10.1093/jee/tow090 PMID: 27122493.
6. Robert CAM, Erb M, Hiltpold I, Hibbard BE, Gaillard MDP, Bilat J, et al. Genetically engineered maize plants reveal distinct costs and benefits of constitutive volatile emissions in the field. Plant Biotechnol J. 2013; 11:628–39. doi: 10.1111/pbi.12053 PMID: 23425633.
7. Robert CAM, Erb M, Hibbard BE, Wade French B, Zwahlen C, Turlings TCJ. A specialist root herbivore reduces plant resistance and uses an induced plant volatile to aggregate in a density-dependent manner. Funct Ecol. 2012; 26:1429–40. doi: 10.1111/j.1365-2435.2012.02030.x.
8. Robert CAM, Erb M, Duployer M, Zwahlen C, Doyen GR, Turlings TCJ. Herbivore-induced plant volatiles mediate host selection by a root herbivore. New Phytol. 2012; 194:1061–9. doi: 10.1111/j.1469-8137.2012.04127.x PMID: 22486361.
9. Bernklau EJ, Bjostad LB. Reinvestigation of host location by western corn rootworm larvae (Coleoptera: Chrysomelidae): CO_2 Is the only volatile attractant. J Econ Entomol. 1998; 91:1331–40. doi: 10.1093/jee/91.6.1331.
