# Supplementary figures and images for: The plant metabolome guides fitness-relevant foraging decisions of a specialist herbivore
Source: PLoS Biol. 2021 Feb 18;19(2):e3001114. doi: 10.1371/journal.pbio.3001114 (PMC7924754; doi:10.1371/journal.pbio.3001114)

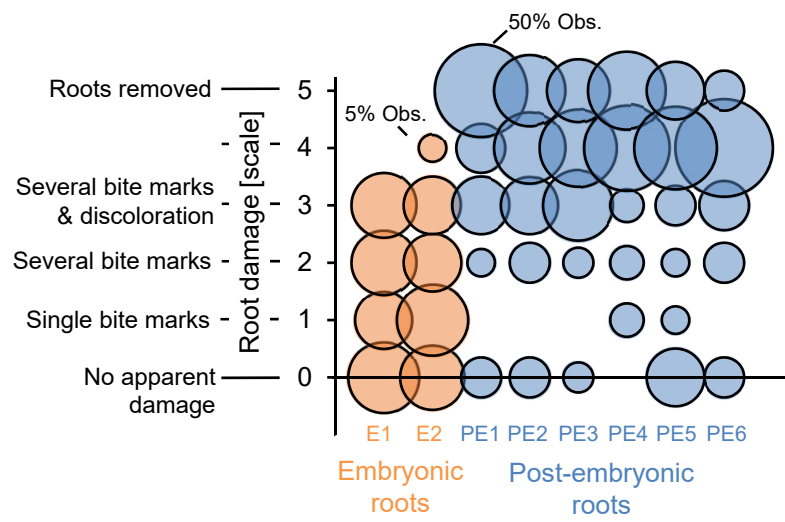

Supplement: S1 Fig — Data are from experiment shown in Fig 1B (n = 20 plants). E1–E2 refer to individual embryonic (E) roots, PE1–PE6 refer to individual postembryonic (PE) roots. Numbers were assigned randomly to embryonic and postembryonic roots within plants. The sizes of the circles are proportional to the relative frequency (% within each root) of the different types of observed damage. Underlying data can be found in S1 Data. (PDF) [file pbio.3001114.s004.pdf]

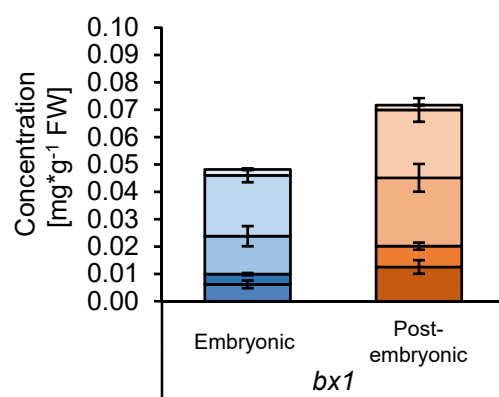

Supplement: S2 Fig — Relative abundances (signal intensities) of identified metabolic features in embryonic and postembryonic roots of WT B73 and bx1 mutant plants (n = 7–10). For sugars and benzoxazinoids, refer to Fig 2. Results of two-way ANOVAs for genotype effects (G), root type effects (R), and their interaction (GxR) are shown for each compound (***p < 0.001, **p < 0.01, *p < 0.05). Error bars denote standard errors of means (SEM). Underlying data can be found in S1 Data. (PDF) [file pbio.3001114.s005.pdf]

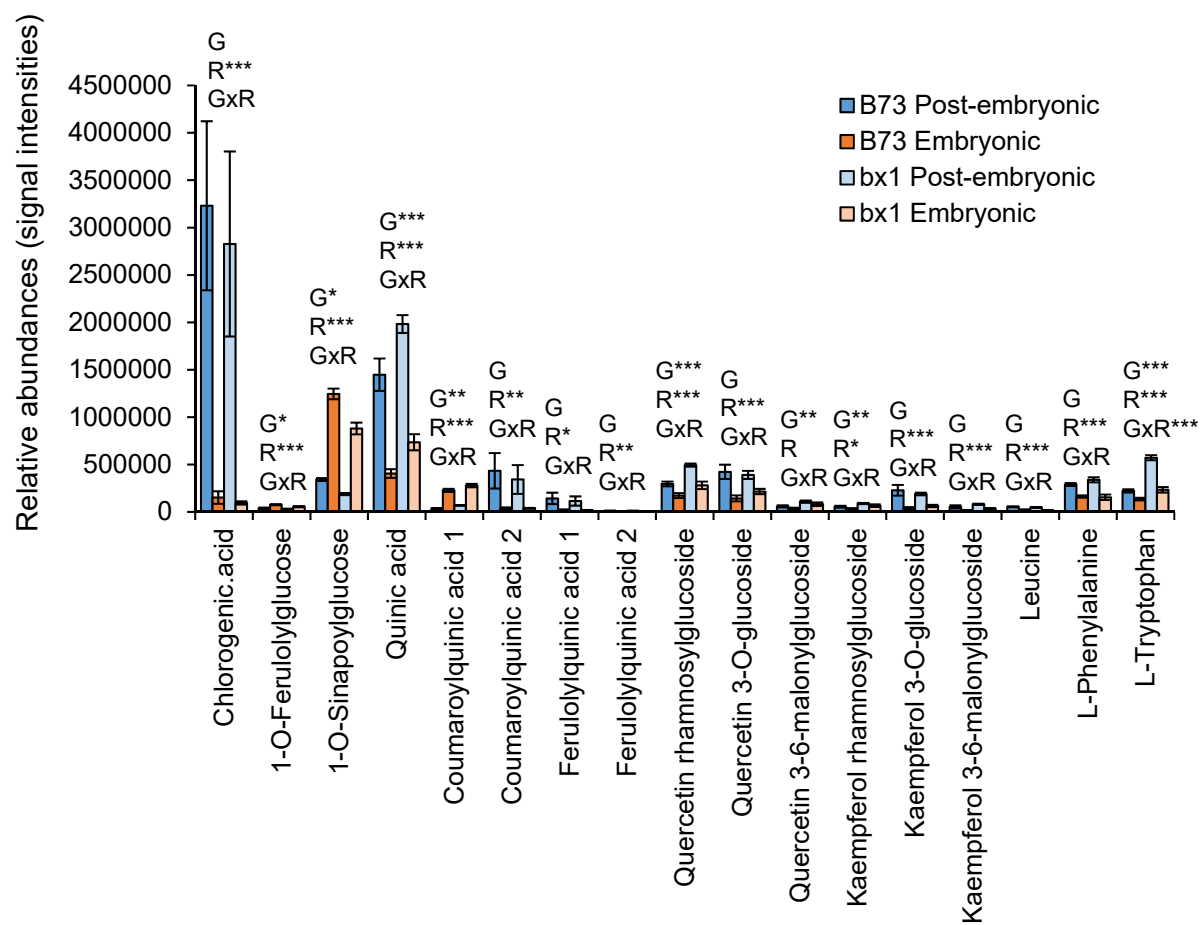

Supplement: S3 Fig — Concentrations of benzoxazinoids in embryonic and postembryonic roots of bx1 mutant plants (n = 11–18). Figure is an expanded panel of Fig 2. No significant differences were found for individual compounds (p > 0.05, Holm–Sidak post hoc tests following two-way ANOVAs). Error bars denote standard errors of means (SEM). Underlying data can be found in S1 Data. (PDF) [file pbio.3001114.s006.pdf]

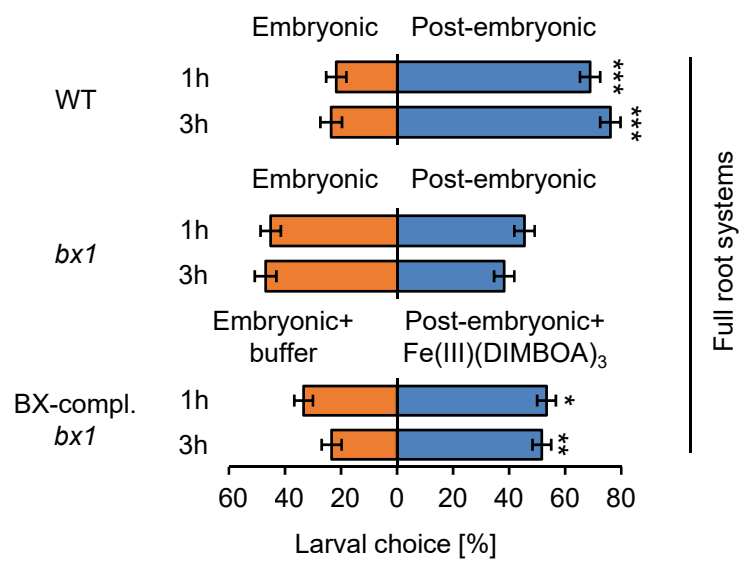

Supplement: S4 Fig — Preference of WT western corn rootworm larvae for embryonic and postembryonic roots of wild type (WT) and bx1 mutant plants in a petri dish assay. For a subset of bx1 mutant root systems, the postembryonic roots were complemented with 200 μl of a Fe(III)(DIMBOA)3 solution at a concentration of 50 μg*ml−1 (v/v) in H2O. Embryonic roots were treated with an equal amount of H2O. Larval choice was recorded after 1 and 3 hours. Asterisks indicate significant preference (***p < 0.001, **p < 0.01, *p < 0.05, FDR-corrected Least Square Mean post hoc tests, n = 10 dishes with 6 larvae each). Underlying data can be found in S1 Data. (PDF) [file pbio.3001114.s007.pdf]

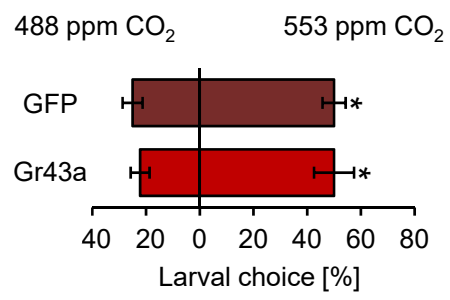

Supplement: S5 Fig — Proportion of control (GFP) or DvvGr43a-silenced (Gr43a) larvae found on each arm of belowground olfactometers. Asterisks indicate significant differences between treatments (*p < 0.05, FDR-corrected Least Square Mean post hoc tests, 6 two-arm olfactometers with 6 larvae each were evaluated, n = 6). Underlying data can be found in S1 Data. (PDF) [file pbio.3001114.s008.pdf]

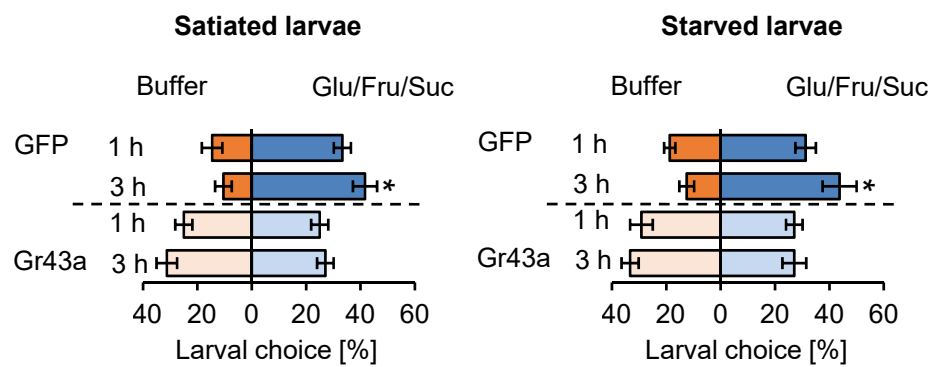

Supplement: S6 Fig — Preference of satiated (left) and starved (right) control or DvvGr43a-silenced larvae for buffer or a glucose, fructose, sucrose mixture on filter discs at different time points (*p < 0.05, FDR-corrected Least Square Mean post hoc tests, 8 petri plates with 6 larvae each were assayed, n = 8). Error bars denote standard errors of means (SEM). Underlying data can be found in S1 Data. (PDF) [file pbio.3001114.s009.pdf]

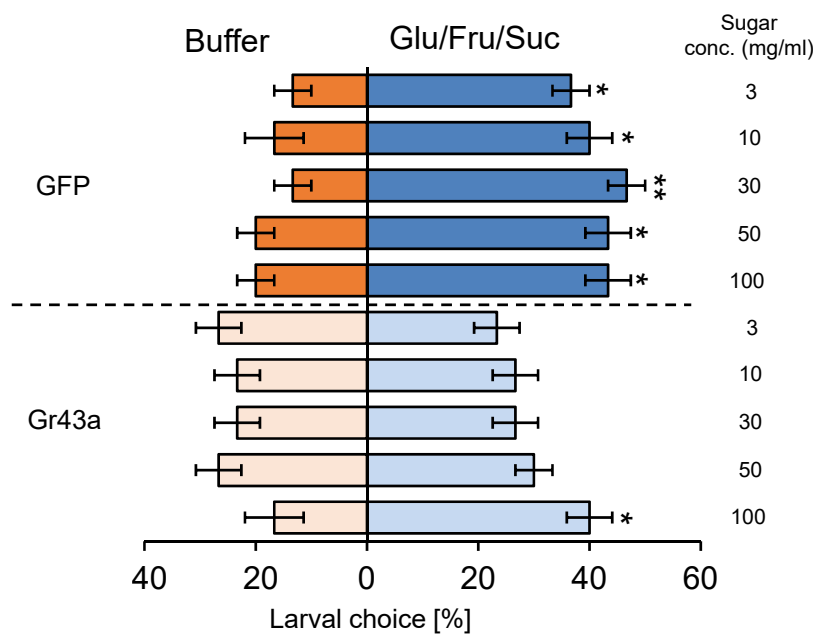

Supplement: S7 Fig — Preference of GFP or DvvGr43a dsRNA fed larvae for glucose, fructose, sucrose mixtures at different concentrations in H2O (v/v) on filter discs 3 hours after the start of the choice experiment. A volume of 10 μl of each individual sugar solution at the indicated concentration was added to the filter discs. Control filter discs were supplied with equal amounts of H2O (*p < 0.05; **p < 0.01; FDR-corrected Least Square Mean post hoc tests, n = 5 dishes with 6 larvae each). Error bars denote standard errors of means (SEM). Underlying data can be found in S1 Data. (PDF) [file pbio.3001114.s010.pdf]

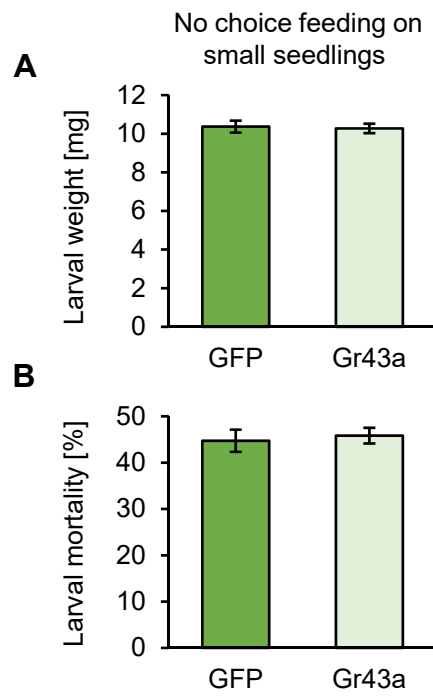

Supplement: S8 Fig — (A) Weight of western corn rootworm larvae fed on GFP and DvvGr43a dsRNA on young maize seedlings that produce embryonic roots only (no-choice setting) for 7 days (n = 40 cups with 9 larvae each). (B) Larval mortality within the same experiment. Underlying data can be found in S1 Data. (PDF) [file pbio.3001114.s011.pdf]
